# Identification of Genes Associated with Chlorophyll Accumulation in Flower Petals

**DOI:** 10.1371/journal.pone.0113738

**Published:** 2014-12-03

**Authors:** Akemi Ohmiya, Masumi Hirashima, Masafumi Yagi, Koji Tanase, Chihiro Yamamizo

**Affiliations:** National Institute of Floricultural Science, National Agriculture and Food Research Organization, Fujimoto 2-1, Tsukuba, Ibaraki 305-8519, Japan; Estación Experimental del Zaidín (CSIC), Spain

## Abstract

Plants have an ability to prevent chlorophyll accumulation, which would mask the bright flower color, in their petals. In contrast, leaves contain substantial amounts of chlorophyll, as it is essential for photosynthesis. The mechanisms of organ-specific chlorophyll accumulation are unknown. To identify factors that determine the chlorophyll content in petals, we compared the expression of genes related to chlorophyll metabolism in different stages of non-green (red and white) petals (very low chlorophyll content), pale-green petals (low chlorophyll content), and leaves (high chlorophyll content) of carnation (*Dianthus caryophyllus* L.). The expression of many genes encoding chlorophyll biosynthesis enzymes, in particular Mg-chelatase, was lower in non-green petals than in leaves. Non-green petals also showed higher expression of genes involved in chlorophyll degradation, including *STAY-GREEN* gene and *pheophytinase*. These data suggest that the absence of chlorophylls in carnation petals may be caused by the low rate of chlorophyll biosynthesis and high rate of degradation. Similar results were obtained by the analysis of *Arabidopsis* microarray data. In carnation, most genes related to chlorophyll biosynthesis were expressed at similar levels in pale-green petals and leaves, whereas the expression of chlorophyll catabolic genes was higher in pale-green petals than in leaves. Therefore, we hypothesize that the difference in chlorophyll content between non-green and pale-green petals is due to different levels of chlorophyll biosynthesis. Our study provides a basis for future molecular and genetic studies on organ-specific chlorophyll accumulation.

## Introduction

Flower differentiation is a complex and highly regulated process that involves changes in shape and color. Flowers develop from florally determined meristems, which in turn proliferate to form the floral organs, including sepals, petals, stamens, and carpels. During these morphological changes, each part of a floral organ shows a distinct pattern of color change that is specific to each plant species. These developmental processes are tightly controlled by multiple genes. In the past decade, analyses of the key regulatory genes have focused primarily on morphological changes and have provided a foundation for understanding the molecular genetic mechanisms controlling the basic pattern of floral architecture [1]–[3]. However, little information is available regarding the molecular mechanisms controlling organ-specific color changes during flower development.

Chlorophylls are Mg^2+^-containing tetrapyrrole compounds responsible for the green color in plants. Because chlorophylls are components of the photosynthetic machinery and are essential for light harvesting and energy transduction, a substantial amount of chlorophyll is present in leaves and stems. Petals of many flowering plants contain chlorophylls at the early developmental stages. As petals mature, their chlorophyll content decreases and other pigments such as anthocyanins, carotenoids, and betalains accumulate [4], [5]. The loss of chlorophylls during petal development is an important trait for flowering plants that enables flowers to be visually distinguished against a background of leaves when the flowers are ready to offer rewards to pollinators.

**Figure 1:**
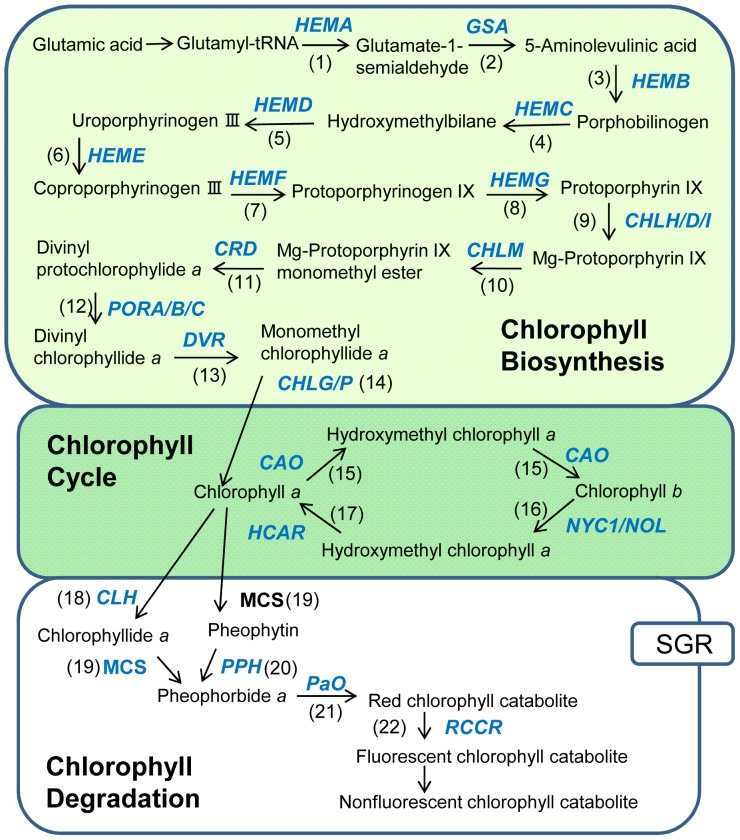
Schematic representation of chlorophyll metabolic pathways in higher plants. Genes (italicized) encode the following enzymes: (1) glutamyl-tRNA reductase; (2) glutamate-1-semialdehyde 2,1-aminotransferase; (3) 5-aminolevulinate dehydrogenase; (4) porphobilinogen deaminase; (5) uroporphyrinogen III synthase; (6) uroporphyrinogen III decarboxylase; (7) coproporphyrinogen III oxidase; (8) protoporphyrinogen oxidase; (9) Mg-chelatase; (10) Mg-protoporphyrin IX methyltransferase; (11) Mg-protoporphyrin IX monomethylester cyclase; (12) protochlorophyllide oxidoreductase; (13) divinyl chlorophyllide *a* 8-vinyl-reductase; (14) chlorophyll synthase; (15) geranylgeranyl-diphosphate reductase (16) chlorophyllide *a* oxygenase; (17) chlorophyll *b* reductase; (18) hydroxymethyl chlorophyll *a* reductase; (19) chlorophyllase; (20) pheophytinase; (21) pheophorbide *a* oxygenase; (22) red chlorophyll catabolite reductase. SGR, STAY-GREEN; MCS, metal-chelating substance.

The chlorophyll metabolic pathway can be divided into three distinct phases (Fig. 1) [6]–[9]: (1) synthesis of chlorophyll *a* from glutamate; (2) interconversion between chlorophyll *a* and *b* (chlorophyll cycle); and (3) degradation of chlorophyll *a* into a non-fluorescent chlorophyll catabolite. The synthesis and activity of enzymes involved in the chlorophyll metabolic pathway are tightly regulated in a time-, development-, and tissue-specific manner. Chlorophylls are associated with chlorophyll-binding proteins of the photosystem I (PSI) and II (PSII) complexes [10], [11] and accumulate in tissues where PSI and PSII are produced. A number of studies have been reported on the genetic elements that control chlorophyll accumulation in photosynthetic tissues [6]–[11]. However, to our knowledge, understanding of the mechanisms that regulate chlorophyll metabolism in petals is completely lacking. Despite that the flowers of most plant species are known to be composed of non-photosynthetic tissues, and the residual photosynthetic activity as well as photosynthesis-related effects of flower petals are areas of research interest [12], [13].

Because numerous genes are involved in various aspects of chlorophyll accumulation, it is difficult to identify a key regulatory factor in petals. In this study, we first carried out a microarray analysis and overview of the expression of genes related to the chlorophyll metabolic pathway during the development of petals and leaves. These data were further validated for selected genes using quantitative real-time PCR (RT-qPCR) analysis. The expression levels of these genes in the pale-green and white petals of several carnation cultivars were also compared. On the basis of these data, we identified the candidate factors controlling chlorophyll accumulation in carnation petals. The microarray data were compared between carnation and *Arabidopsis thaliana*, and showed common features in the control of chlorophyll levels in the two species.

## Materials and Methods

### Plant materials

Carnation (*Dianthus caryophyllus* L.) cultivars were grown under natural daylight conditions in a greenhouse at the National Institute of Floricultural Science (Tsukuba, Ibaraki, Japan). Petals (stages 1 to 4; Fig. S1 in File S1) and leaves (young and mature leaves; Fig. S1 in File S1) were harvested at different stages from April 30 to May 9, 2010. Sunshine hours and average temperature during flower development (April 1 to May 9) were 257 h and 13.4°C, respectively. Samples were immediately frozen in liquid nitrogen, and stored at −80°C until use. Carnation petals consist of distinct lower and upper parts (Fig. S1 in File S1). The lower part is narrow and pale green; the upper part is wide and exhibits the color characteristic of the cultivar. The upper part of petals were used for chlorophyll content and gene expression analyses.

### Quantitative real-time PCR analysis (RT-qPCR)

RT-qPCR was performed as described previously [14]. The analyses were performed in triplicate and the data were normalized to mRNA levels of *actin* of each sample. For RT-qPCR standard curve assays, cDNA for each gene was amplified by RT-PCR, cloned into a pCR2.1 vector (Invitrogen, Carlsbad, CA, USA). Primers (Table S1 in File S1) used for the cloning were designed based on the expressed sequence tag (EST) sequences [15]. Cloned cDNAs were sequenced and primers for RT-qPCR were designed based on the sequences (Table S2 in File S1). No ESTs corresponding to *chlorophyllase* (*CLH*), *pheophytinase* (*PPH*), and *Rubisco large subunit* (*RbcL*) were found. Instead, partial cDNAs encoding these enzymes were cloned by RT-PCR using degenerate primers (Table S1 in File S1) and sequenced. The GenBank accession numbers for *CLH*, *PPH*, and *RbcL* are AB839760, AB839759, and AB839761, respectively.

### Chlorophyll analysis

Tissues were ground into powder in liquid nitrogen and extracted with acetone. The samples were centrifuged at 10,000× *g* for 10 min, and the supernatants (80 µl) were mixed with 20 µl of water. Pigments were analyzed by high-performance liquid chromatography (HPLC) using a reversed-phase column (Symmetry C8, 150×4.6 mm; Waters, Milford, MA, USA) according to Zapata et al. [16]. The analysis was performed in triplicate.

### Statistical analysis

The significance of differences in chlorophyll content and gene expression was analyzed by Tukey's honestly significant difference tests (*P*<0.05) for multiple comparisons using SPSS Statistics 19 (IBM, New York, USA). Pairwise comparisons were performed using Student's *t*-tests (*P*<0.05). To examine the relationship between chlorophyll content and gene expression level, the Pearson correlation coefficient was calculated.

## Results

### Chlorophyll content in petals and leaves

**Figure 2:**
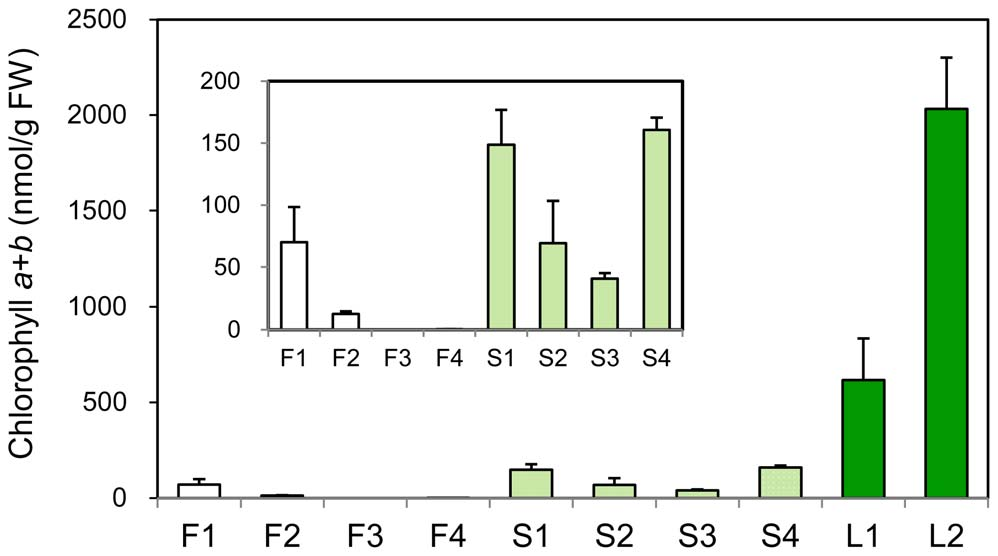
Changes in chlorophyll content during development of Francesco petals, Seychelles petals, and Francesco leaves; 1 to 4, petals at stages 1 to 4 (F, Francesco; S, Seychelles); L1 and L2, young (L1) and mature (L2) Francesco leaves. Mean values (± SD) are shown (*n* = 3). A graph with an expanded ordinate is shown in the inset.

In the red-flowered cultivar Francesco, small amounts of chlorophyll accumulated at the early developmental stages (1 and 2) (Fig. 2). At the late stages (3 and 4), chlorophyll content decreased to extremely low levels. Larger quantities of chlorophyll were detected in petals of the pale-green-flowered cultivar Seychelles than in Francesco (Fig. S2 in File S1); chlorophyll content decreased at stages 2 and 3, but recovers at stage 4 to the levels similar to those at stage 1. Chlorophyll content in petals of Francesco and Seychelles at stage 4 was approximately 0.02% and 7.9% of that in mature leaves of Francesco.

### Analysis of chlorophyll-related gene expression

#### Chlorophyll biosynthesis

A carnation custom oligonucleotide array constructed in our previous study [14] covered the majority of genes involved in chlorophyll metabolism and accumulation. In this study, expression profiles of genes related to chlorophyll metabolism were extracted from the microarray data and hierarchical clustering analysis was performed based on these profiles (Fig. S3 in File S1). Hierarchical clustering analysis of chlorophyll biosynthesis genes revealed three distinct expression patterns (Fig. S3A in File S1). Genes in group 1, *CHLH* and *CHLI* [encoding Mg-protoporphyrin IX chelatase (Mg-chelatase) subunits], *HEMA* (encoding glutamyl-tRNA reductase), and *CHLM* [encoding Mg-protoporphyrin IX methyltransferase (MgPMT)], showed extremely low expression in Francesco petals throughout the developmental stages. Genes in group 2, *DVR* (encoding divinyl chlorophyllide *a* 8-vinyl-reductase), *HEME* (encoding uroporphyrinogen III decarboxylase), *HEMB* (encoding 5-aminolevulinate dehydratase), and *CHLG* (encoding chlorophyll synthase), were constitutively expressed in all tissues examined. Genes in group 3, *CHLD* (encoding uroporphyrinogen III synthase), *HEMC* (encoding hydroxymethylbilane synthase), *POR* (encoding protochlorophyllide oxidoreductase), *GSA* (encoding glutamate-1-semialdehyde 2,1-aminotransferase), *HEMF* (encoding coproporphyrinogen III oxidase), and *HEMG* (encoding protoporphyrinogen III oxidase), were highly expressed in Francesco petals at stage 1.

**Figure 3:**
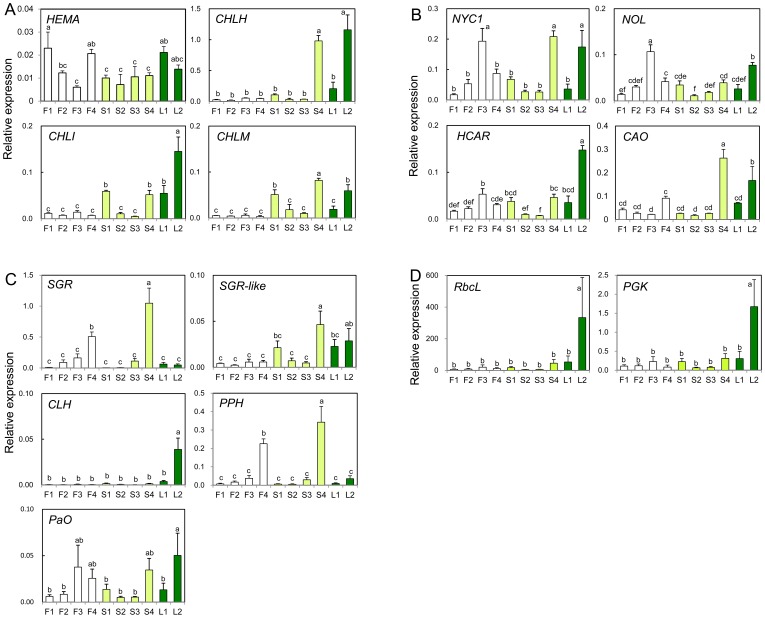
Relative expression of genes related to chlorophyll biosynthesis (A), chlorophyll cycle (B), chlorophyll degradation (C), and photosynthesis (D). Mean values (± SD) are shown (*n* = 3). Different letters indicate significant differences (Tukey's honestly significant difference test, *P*<0.05). Designations of petal and leaf development stages and cultivars are as in Fig. 2. *CAO*, chlorophyllide *a* oxygenase; *CHLH*/*CHLI*, Mg-protoporphyrin IX chelatase subunits; *CHLM*, Mg-protoporphyrin IX methyltransferase; *CLH*, chlorophyllase; *HCAR*, hydroxymethyl chlorophyll *a* reductase; *HEMA*, glutamyl-tRNA reductase; *NYC1*/*NOL*, chlorophyll *b* reductase; *SGR*, STAY-GREEN; *PaO*, pheophorbide *a* oxygenase; *PGK*, phosphoglycerate kinase; *PPH*, pheophytinase; *RbcL*, Rubisco large subunit.

Because the expression patterns of group 1 genes were well correlated with chlorophyll content, we hypothesize that these genes may play an important role in determining chlorophyll content in petals. Expression of group 1 genes was further analyzed by RT-qPCR. As observed in the microarray analysis, the levels of *CHLH*, *CHLI*, and *CHLM* transcripts were extremely low in Francesco petals at all stages (Fig. 3A). In Seychelles petals, expression of *CHLH*, *CHLI*, and *CHLM* decreased between stages 1 to 3 and then increased at stage 4. The expression levels of these three transcripts at stage 4 in Seychelles petals were significantly higher than those in Francesco petals and were similar to those in Francesco leaves. In contrast, expression of *HEMA* was significantly higher in Francesco petals than in Seychelles petals, whereas the level of *HEMA* expression was comparatively lower according to the microarray analysis results (Fig. S2A in File S1). This result may be partly due to the fact that the sequences used for microarray probes and those used for RT-qPCR primers were different. Among the group 1 genes, changes in the expression levels of *CHLI* and *CHLH* during development of the petals and leaves were well correlated with chlorophyll content [Pearson correlation coefficients *r* = 0.92 (*p*<0.01) and *r* = 0.74 (*p*<0.05), respectively; Table S3 in File S1].

#### Chlorophyll cycle

RT-qPCR analysis of genes related to the chlorophyll cycle (Fig. 3B) showed similar expression patterns to those observed in the microarray analysis (Fig. S3B in File S1). The expression levels of *NYC1* (*Non-Yellow Coloring 1*), *NOL* (*NYC-one like*), and *HCAR* (encoding hydroxymethyl chlorophyll *a* reductase) were low at stages 2 and 3 in Seychelles petals but were significantly higher at stage 3 than at the other stages in Francesco petals. The levels of *NYC1* transcripts in mature leaves were similar to those in Francesco petals at stage 3. Expression of *HCAR* was significantly lower in petals than in mature leaves throughout development. The expression of *CAO* (encoding chlorophyll synthase) significantly increased in Seychelles petals at stage 4. Among the genes involved in the chlorophyll cycle, only *HCAR* showed a significant correlation with chlorophyll content [*r* = 0.91 (*p*<0.01); Table S3 in File S1].

#### Chlorophyll degradation

The expression patterns of chlorophyll degradation genes determined by RT-qPCR were similar to those determined by microarray (Fig. S3C in File S1). The expression of *SGR* and *PPH* increased as petals matured in both Francesco and Seychelles and was significantly higher in stage 4 petals than in Francesco leaves (Fig. 3C). The expression pattern of *SGR-like* was similar to that of *CHLH*, *CHLI*, and *CHLM*: its levels were low at all stages in Francesco petals and at stages 2 and 3 in Seychelles petals. The expression pattern of *PaO* was similar to that of *NYC1/NOL* and *HCAR*, with the highest levels at stages 3 and 4 in Francesco and Seychelles petals, respectively. The expression of *CLH* was significantly higher in mature leaves than in petals of both cultivars throughout development and positively correlated with chlorophyll content [*r* = 0.98 (*p*<0.01); Table S3 in File S1].

#### Photosynthesis-related genes

Hierarchical clustering analysis of genes encoding photosynthesis-related proteins revealed two distinct expression patterns (Fig. S3D in File S1). Genes in group 1, including core proteins of PSI (PsaA) and PSII (PsbB and PsbD), were constitutively expressed in all tissues examined. These genes also showed relatively high expression in stage 1 petals in Francesco. Genes in group 2 primarily encoded antenna proteins of the light-harvesting complexes (LHCs) of PSI (Lhca1 and Lhca4) and PSII (Lhcb3 and Lhcb4.2), and showed extremely low expression in Francesco petals; expression levels of these genes in Seychelles petals at stage 4 were similar to those in Francesco leaves. The levels of transcripts encoding phosphoglycerate kinase (PGK), a key enzyme in the Calvin cycle, were lower in Francesco petals than in leaves and Seychelles petals.

Unexpectedly, no transcripts for Rubisco, the key enzyme in the photosynthesis, were found in the carnation EST database [15]. Therefore, we obtained partial cDNA encoding RbcL by RT-PCR with degenerate primers, and performed RT-qPCR analysis. The expression of both *RbcL* and *PGK* in mature Francesco leaves was significantly higher than that in petals throughout development (Fig. 3D). The expression of both genes were positively correlated with chlorophyll content [*r* = 0.98 (*p*<0.01) and *r* = 0.97 (*p*<0.01), respectively; Table S3 in File S1].

### Comparison of gene expression between green and white petals

**Figure 4:**
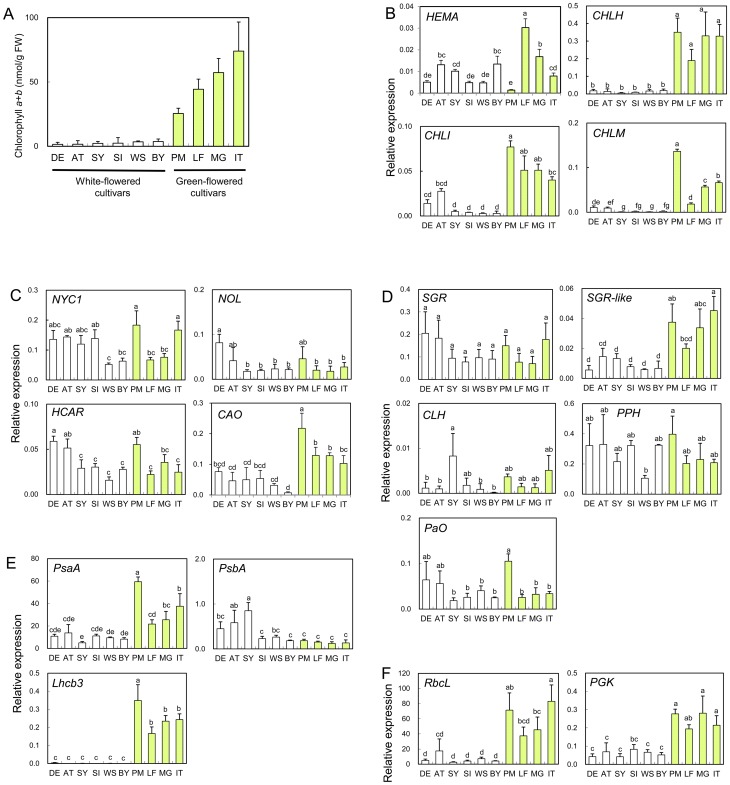
Relative expression of chlorophyll-related genes in petals of white- and pale-green-flowered carnation cultivars. Chlorophyll content (A), and expression of genes related to chlorophyll biosynthesis (B), chlorophyll cycle (C), chlorophyll degradation (D), and photosynthesis (E). Mean values (± SD) are shown (*n* = 3). Upper parts of petals from fully opened flowers (stage 4) were used for the analysis. Different letters indicate significant differences (Tukey's honestly significant difference test, *P*<0.05). Gene abbreviations are as in Fig. 3. White-flowered cultivars: WS, White Sim; AT, Atlantis; BY, Byakko; SI, Siberia; SY, Shirayuki; DE, Delphi. Pale-green-flowered cultivars: PM, Prado Mint; LF, Le France; IT, Ice Tea; MG, Martha Green.

Chlorophyll content in stage 4 petals of white-flowered cultivars was less than 4 nmol/g fresh weight (FW), whereas it was 25–74 nmol/gFW in pale-green-flowered cultivars (Fig. 4A). The expression of selected genes in white and pale-green petals was compared by RT-qPCR analysis.

Among group 1 genes in the chlorophyll biosynthesis (Fig. S3 in File S1), *CHLI* and *CHLH* showed positive correlation with chlorophyll content [*r* = 0.66 (*p*<0.05) and *r* = 0.87 (*p*<0.01), respectively; Fig. 4B, Table S4 in File S1]. There was considerable variability among cultivars in the expression levels of genes related to the chlorophyll cycle (*NYC1*, *NOL*, *CAO* and *HCAR*; Fig. 4C) and chlorophyll degradation (*SGR*, *CLH*, *PPH*, and *PaO*; Fig. 4D), but there was no significant correlation between transcript levels and chlorophyll content (Table S4 in File S1). In contrast, the levels of *SGR-like* transcripts were significantly higher in pale-green petals than in white petals [*r* = 0.87 (*p*<0.01); Figs. 4D, Table S4 in File S1].

The expression of photosynthesis-related genes, such as *Lhcb3*, *RbcL*, and *PGK*, was positively correlated with chlorophyll content [*r* = 0.79 (*p*<0.01), *r* = 0.85 (*p*<0.01), and *r* = 0.82 (*p*<0.01), respectively; Fig. 4E and F, Table S4 in File S1]. Expression of *Lhcb3* was particularly strongly suppressed in white petals. Some white-flowered cultivars and pale-green-flowered cultivars showed significantly higher levels of *PsbA* and *PsaA* transcripts, respectively, but there was no significant correlation between their expression levels and chlorophyll content (Fig. 4A, Table S4 in File S1).

### Senescence-induced changes in *SGR* gene expression

**Figure 5:**
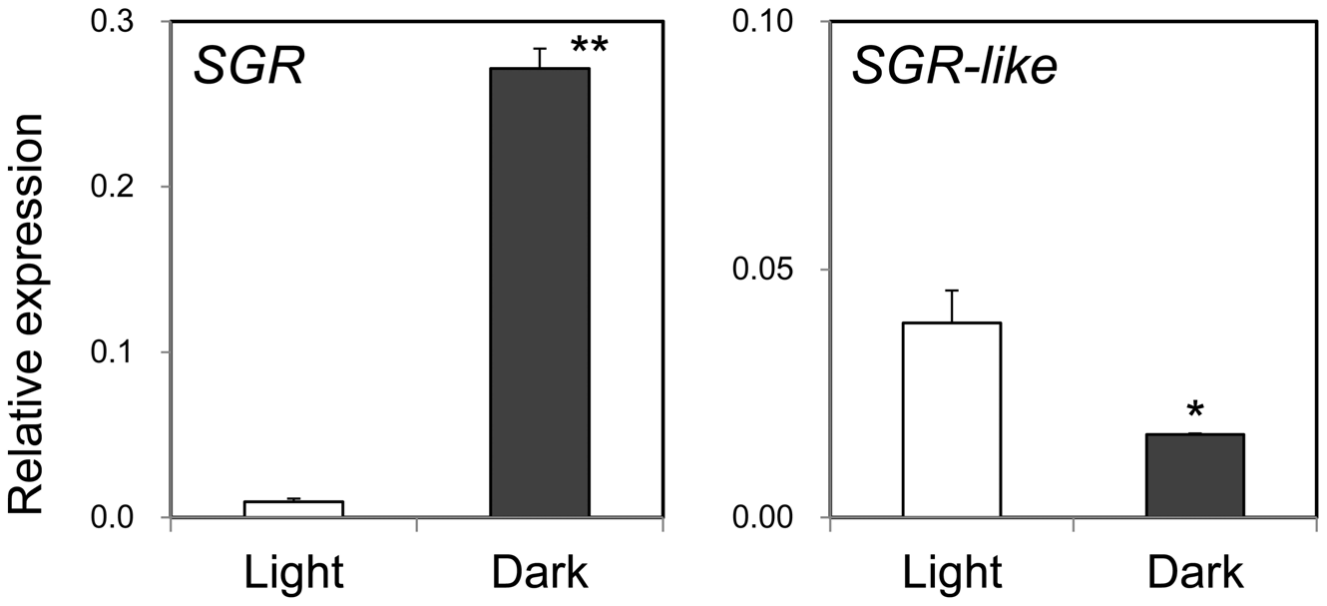
Comparison of *SGR* and *SGR-like* gene expression in Francesco leaves grown under natural daylight or in the dark (covered with aluminum foil) for one week to induce senescence. Mean values (± SD) are shown (*n* = 3). Statistical differences were analyzed by Student's *t*-test (*P<0.05; **P<0.01).

We found two *SGR* ESTs, *SGR* and *SGR-like*, in the carnation EST database [15], with 49% similarity of deduced amino acid sequences. Phylogenetic analysis showed that the deduced amino acid sequence of carnation SGR is closely related to that of *Arabidopsis* SGR1 and SGR2, and that carnation SGR-like is in the same clade as *Arabidopsis* SGR-like (Fig. S4 in File S1). The expression of *SGR* and *SGR-like* was compared between control (light-grown) leaves and leaves exposed to darkness for 1 week to induce senescence (Fig. 5). *SGR* expression in leaves exposed to darkness was 28-fold higher than that in the control leaves, indicating that expression of this gene was strongly induced by leaf senescence. In contrast, the expression of *SGR-like* in control leaves was 2.3-fold higher than that in dark-grown leaves.

### Expression of chlorophyll metabolism–related genes in *Arabidopsis*

In *Arabidopsis*, most of the genes involved in chlorophyll biosynthesis showed lower expression levels in petals than in leaves (Fig. S5A in File S1). In particular, the levels of *DVR*, *PORC*, *PORB*, and *GSA2* transcripts in petals at stage 15 (P2) were extremely low. Among genes involved in the chlorophyll cycle, the level of *CAO* transcripts was lower, whereas *NYC1* expression was higher, in petals at stage 15 than in leaves (Fig. S5B in File S1). Among the chlorophyll catabolic genes, the expression levels of *PPH*, *PaO*, and *SGR1* were higher, whereas those of *SGR-like* and *RCCR* were lower, in petals at stage 15 than in leaves (Fig. S5C in File S1).

## Discussion

To clarify the key factors that determine differential levels of chlorophyll accumulation between leaves and petals, we compared the expression of genes involved in chlorophyll metabolism and detected a difference in expression levels of genes involved in chlorophyll degradation in carnation. In particular, the levels of *PPH* and *SGR* transcripts were differed significantly between leaves and petals. PPH has phytol-cleavage activity, accepts pheophytin *a* as substrates, and converts these substrates into the phytol-free pigment pheophorbide *a* [17] (Fig. 1). In *Arabidopsis* leaves, *PPH* expression was induced by darkness when chlorophyll degradation was accelerated [17]. The loss of PPH activity causes a stay-green phenotype, which shows retarded chlorophyll degradation during senescence. These studies indicate that PPH plays an important role in chlorophyll degradation in leaves. However, it is not known whether PPH is also involved in chlorophyll degradation in other tissues including petals. CLH is another phytol-cleaving enzyme and produces chlorophyllide *a* from chlorophyll *a* (Fig. 1); however, its involvement in chlorophyll degradation is still a matter of debate. The two *Arabidopsis* CLHs, AtCLH1 and AtCLH2, are localized not in the chloroplasts (where chlorophylls accumulate) but in the cytosol [18]. In a loss-of-function CLH mutant, chlorophyll degradation during leaf senescence is not affected, suggesting that CLHs are dispensable for chlorophyll breakdown [18]–[20]. In contrast, CLH activity is tightly associated with chlorophyll breakdown during citrus fruit ripening and overexpression of citrus *CLH* results in enhanced chlorophyll breakdown [21]. In carnation, *PPH* expression strongly increased in petals at stage 4 and was significantly higher than that in mature leaves, whereas *CLH* expression was severely suppressed at all stages of petal development compared with mature leaves. Thus, we assume that PPH, but not CLH, is important for phytol cleavage in the chlorophyll degradation pathway in carnation petals.

*SGR* has been identified as a gene responsible for stay-green phenotypes in rice and *Arabidopsis* [22], [23]. Although SGR has no chlorophyll catalytic activity, several lines of evidence indicate that this protein is involved in the initiation of chlorophyll degradation via destabilization of protein–pigment complexes in the thylakoid membranes [24], [25]. SGR is also responsible for the stay-green phenotypes in tomato and pepper fruit, in which chlorophyll degradation normally occurs at the onset of fruit ripening [26]. We showed that the expression of *SGR* is significantly higher in stage 4 petals than in leaves in both carnation and *Arabidopsis*. These results suggest that SGR is also involved in chlorophyll degradation in non-photosynthetic tissues and may partially contribute to the absence of chlorophylls in petals. However, analysis of the Pearson correlation coefficient showed that there was no correlation between *SGR* and *PPH* expression levels and chlorophyll content, suggesting that the amount of chlorophyll in petals is determined not only by the degradation rate but also by other unknown factors.

Two or more homologous SGR-encoding genes with organ-specific expression and differential developmental regulation have been detected in many plants [26], [27]. In *Arabidopsis*, expression of *SGR1* (At4g22920) increases at the onset of leaf senescence, whereas *SGR-like* (At1g44000) is highly expressed in the cotyledons and developing leaves. Our phylogenetic analysis showed that carnation SGR belongs to the same clade as *Arabidopsis* SGR1 and pea SGR, which are involved in chlorophyll degradation [23], [28]. The expression of carnation *SGR* was drastically increased by dark-induced senescence. Therefore, we assume that carnation SGR is more closely related to *Arabidopsis* SGR1 and may be involved in chlorophyll degradation in petals and senescent leaves. Carnation SGR-like belongs to the same clade as *Arabidopsis* SGR-like, and its gene was highly expressed in developing leaves. In addition, SGR-like expression decreased under dark condition. This expression pattern is similar to that observed in *SGR-like in Arabidopsis* [29]. Recently, Sakuraba et al. [30] demonstrated that *Arabidopsis* SGR-like plays an important role in the early phase of chlorophyll degradation. Although the genes encoding SGR-like proteins have been identified in various organisms (Fig. S4 in File S1), the functions of these proteins in petals remain unknown. Significantly higher *SGR-like* expression in pale-green petals than in white petals led us to speculate that SGR-like contributes to the increase in chlorophyll content in carnation petals.

In addition to the mutations in *SGR* and *PPH* described above, mutations in *NYC1*, *NOL*, *HCAR*, and *PaO* also cause the stay-green phenotype in *Arabidopsis* [31]–[34]. Analysis of the expression levels of these stay-green genes in white and pale-green petals showed that there was no correlation between chlorophyll content and expression levels, suggesting that the pale-green phenotype of carnation petals was not caused by the suppression of stay-green gene expression. It is interesting to note that the level of *HCAR* transcripts showed positive correlation with chlorophyll content during development in the petals and leaves of carnation. Similarly, in *Arabidopsis*, HCAR expression is up-regulated in greening seedlings [34]. These results suggest that HCAR plays a major role in the chlorophyll cycle during development.

The expression of genes involved in chlorophyll degradation was similar in non-green and green carnation petals. In contrast, the expression profiles of chlorophyll biosynthesis genes were lower in non-green petals than in pale-green petals. The most prominent feature was the extremely low expression of genes encoding Mg-chelatase subunits (encoded by *CHLH* and *CHLI*) in non-green petals. In many plants, the loss of Mg-chelatase activity leads to the white leaf phenotype, indicating that these enzymes play a key role in regulation of chlorophyll biosynthesis [35]–[37]. We assume that chlorophyll biosynthesis may be lower in non-green petals than in leaves and pale-green petals because of the extremely low levels of Mg-chelatase activity.

**Figure 6:**
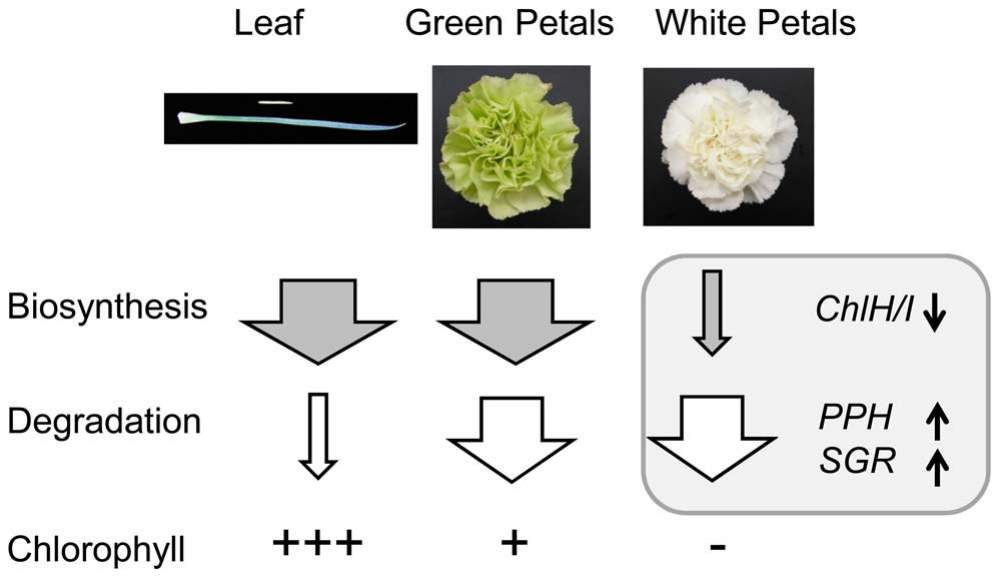
Possible mechanism controlling chlorophyll content in petals and leaves. Arrow width indicates the extent of activity of each pathway. *CHLH*/*CHLI*, Mg-protoporphyrin IX chelatase subunits; *PPH*, pheophytinase; *SGR*, STAY-GREEN.

Pale-green petals showed higher expression of chlorophyll biosynthesis genes than non-green petals, but similar expression of genes involved in chlorophyll degradation. Therefore, we assume that differences in chlorophyll content between non-green and pale-green petals can be attributed to different levels of chlorophyll biosynthesis (Fig. 6). Pale-green color of petals can be expected to be dominant over white if flower color is determined by chlorophyll biosynthesis capacity. To test this hypothesis, we performed crosses between the white-flowered cultivar Shirayuki (female) and the pale-green-flowered cultivar Seychelles (male). All the F_1_ progenies showed pale-green petals (Fig. S6 in File S1), indicating the dominance of this color.

Chlorophylls and carotenoids form the light-harvesting antennae within the thylakoids, in which carotenoids provides energy transfer to reaction centers. In addition, carotenoids are involved in photoprotection mechanisms [38]. Therefore, biosynthesis of chlorophyll and carotenoids in the chloroplast must be coordinated. In carnation, the carotenoid profile in pale-green petals is similar to that in leaves, suggesting that carotenoids are components of the light-harvesting antennae in petal chloroplasts [14]. The low levels of carotenoids in non-green petals are caused not by enzymatic degradation but rather by low rates of carotenoid biosynthesis, as suggested by the extremely low expression of carotenogenic genes (in particular, *phytoene synthase* and *lycopene ε-cyclase*) in non-green petals. It is of particular interest whether transcription of genes that encode the key enzymes of chlorophyll and carotenoid biosynthesis is regulated by a common mechanism.

Chlorophylls are noncovalently associated with chlorophyll-binding proteins located in the thylakoid membrane [11]. Chlorophylls and chlorophyll-binding proteins may be synthesized coordinately because free chlorophyll would enhance photo-oxidative damage in the cells [39]–[42]. In carnation petals, the absence of chlorophylls was tightly associated with the low expression of genes encoding several chlorophyll-binding proteins, including LHC antenna proteins, whereas expression of the core proteins, such as PsaA and PsbA, was less affected. A simultaneous reduction of *CHLH*, *CHLI*, *CHLM*, and *Lhcb* transcripts was observed, suggesting that Mg-chelatase and/or MgPMT activity may affect LHC proteins at the transcriptional level. In *Arabidopsis*, a *CHLM* mutation was found to suppress *Lhcb* expression [43]. It remains to be established whether MgPMT serves as a signaling molecule to control the expression of nuclear-encoded photosynthesis genes.

Chlorophyll-containing flowers, including carnation, have photosynthetically active chloroplasts that contribute to the flowers' supply of carbohydrates [12], [44]–[46]. During early stages of petal development, carnation petals contain substantial levels of the large and small subunits of Rubisco, which decline as the petals mature [44]. Our data showing that levels of *RbcL* and *PGK* transcripts are correlated with chlorophyll content in carnation petals indicate that their transcription is coordinated with chlorophyll accumulation.

In conclusion, based on our analysis of gene expression and chlorophyll content, we suggest that low rate of chlorophyll biosynthesis and high rate of chlorophyll degradation lead to the absence of chlorophylls in non-green carnation petals (Fig. 6). Higher rate of chlorophyll biosynthesis in pale-green petals may result from the loss of suppressors of this process.

## Supporting Information

File S1 **Figures S1–S6 and Tables S1–S4.** **Figure S1.** Photographs of carnation flowers and leaves used in this study. **Figure S2.** HPLC chromatograms of chlorophyll extract from stage 4 petals. **Figure S3.** Overview of expression profiles of genes related to chlorophyll biosynthesis, chlorophyll cycle, chlorophyll degradation, and photosynthesis in petals and leaves of carnation. **Figure S4.** Phylogenetic tree of SGR proteins. **Figure S5.** Expression profiles of genes related to chlorophyll biosynthesis, chlorophyll cycle, chlorophyll degradation, and photosynthesis in petals and leaves of *Arabidopsis*. **Figure S6.** F_1_ progenies obtained by crosses between the white-flowered carnation cultivar Shirayuki (female) and the pale-green-flowered cultivar Seychelles (male). **Table S1.** Degenerate primers used for cloning. **Table S2.** Primers used for RT-qPCR analysis. **Table S3.** Correlation between chlorophyll content and gene expression level in petals and leaves. **Table S4.** Correlation between chlorophyll content and gene expression level in white and green petals.
(PDF)
