# Supplementary material for: Identification of Genes Associated with Chlorophyll Accumulation in Flower Petals
Source: PLoS One. 2014 Dec 3;9(12):e113738. doi: 10.1371/journal.pone.0113738 (PMC4254739; doi:10.1371/journal.pone.0113738)
Supplement: File S1 — Figures S1–S6 and Tables S1–S4. Figure S1. Photographs of carnation flowers and leaves used in this study. Figure S2. HPLC chromatograms of chlorophyll extract from stage 4 petals. Figure S3. Overview of expression profiles of genes related to chlorophyll biosynthesis, chlorophyll cycle, chlorophyll degradation, and photosynthesis in petals and leaves of carnation. Figure S4. Phylogenetic tree of SGR proteins. Figure S5. Expression profiles of genes related to chlorophyll biosynthesis, chlorophyll cycle, chlorophyll degradation, and photosynthesis in petals and leaves of Arabidopsis. Figure S6. F1 progenies obtained by crosses between the white-flowered carnation cultivar Shirayuki (female) and the pale-green-flowered cultivar Seychelles (male). Table S1. Degenerate primers used for cloning. Table S2. Primers used for RT-qPCR analysis. Table S3. Correlation between chlorophyll content and gene expression level in petals and leaves. Table S4. Correlation between chlorophyll content and gene expression level in white and green petals. (PDF) [file pone.0113738.s001.pdf]

## Identification of genes associated with chlorophyll accumulation in flower petals.

**Akemi Ohmiya*, Masumi Hirashima, Masafumi Yagi, Koji Tanase, and Chihiro Yamamizo**

Supporting Information

Fig. S1. Photographs of carnation flowers and leaves used in this study. Flowers and petals at stages 1 to 4 of Francesco (F1–F4;A) and Seychelles (S1–S4;B). Young (L1) and mature (L2) leaves of Francesco (C).

Fig. S2. HPLC analysis of chlorophyll extract from stage 4 petals. HPLC chromatogram obtained at 650 nm (A) and absorption spectra of peak1(chlorophyll *a*) and peak2 (chlorophyll *b*) (B).

Fig. S3. Overview of expression profiles of genes related to chlorophyll biosynthesis (A), chlorophyll cycle (B), chlorophyll degradation (C), and photosynthesis (D) in petals and leaves of carnation. Stages are designated as in Fig. S1. Custom oligonucleotide array was constructed and microarray analysis was performed as previously described (Ohmiya et al. 2013[14]). The microarray data is clustered with respect to genes using the GeneSpring XII (Agilent) and dendrograms are shown in the left side of the heatmap. Color indicates normalized signal intensities as shown in the bar below the heatmap.

EST IDs of contig sequences for the analyzed genes are as follows: *CAO* (FX298394), *CHLD* (FX333620), *CHLG* (FX315257), *CHLH* (FX298164), *CHLI* (FX298942), *CHLM* (FX299996), *DVR* (FX296796), *HCAR* (FX316722), *HEMA1* (FX299213), *HEMB1* (FX310043), *HEMB2* (FX300138), *HEMC* (FX298880), *HEME1* (FX322585), *HEME2* (FX297078), *HEMF1* (FX297405), *HEMF2* (FX297404), *HEMG2* (FX322072), *Lhca1* (FX296589), *Lhca4* (FX296782), *Lhcb2.4* (FX310054), *Lhcb3* (FX297100), *Lhcb4.2* (FX296541), *NOL* (FX297130), *NYC1* (FX306746), *PaO* (FX299444), *PGK* (FX297239), *POR* (FX329216), *PsaA* (FX304959), *PsbA* (FX317361), *PsbB* (FX306529), *PsbD* (FX312224), *SGR* (FX315502), and *SGR-like* (FX300055). GenBank accession numbers of *PPH* and *GSA* are AB839759 and AB839944, respectively.

Fig. S4. Phylogenetic tree of SGR proteins. Sequences were retrieved from the GenBank database, and their phylogenetic relationship was deduced by using the neighbor-joining method. Numbers at branch points indicate bootstrap values (1,000 replicates). Scale bar, 0.1 amino acid substitutions per site. Protein accession numbers are as follows: AAW82962 (*Arabidopsis thaliana*, AtSGR1), AAU05981 (AtSGR2), AAM14392 (AtSGR-like), YP_001391480 (*Clostridium botulinum*, CbSGR), AAW82959 (*Glycine max*, GmSGR1), AAW82960 (GmSGR2), AAW82955 (*Hordeum vulgare*, HvSGR), AAY98500 (*Lycopersicon esculentum*, LeSGR), AAW82954 (*Oryza sativa*, OsSGR1), BAF16284 (OsSGR2), CAE05787 (OsSGR3), CAL56489 (*Ostreococcus tauri*, OtSGR), EDQ70701 (*Physcomitrella patens*, PpSGR1), EDQ62217 (PpSGR2), EDQ81746 (PpSGR-like), BAF76352 (*Pisum sativum*, PsSGR-JI2775), CAP04954 (PsSGR-JI4), AAW82958 (*Sorghum bicolor*, SbSGR), AAW82956 (*Zea mays*, ZmSGR1), and AAW82957 (ZmSGR2).

Fig. S5. Expression profiles of genes related to chlorophyll biosynthesis (A), chlorophyll cycle (B), chlorophyll degradation (C), and photosynthesis (D) in petals and leaves of *Arabidopsis*. The microarray data were obtained from the AtGenExpress database (available from TAIR: http://www.arabidopsis.org). P1, flower stage 12, petal (ATGE_35); P2, flower stage 15, petal (ATGE_42); L, leaf (ATGE_5). The microarray data is clustered with respect to genes using the GeneSpring XII (Agilent) and dendrograms are shown in the left side of the heatmap. Color indicates normalized signal intensities as shown in the bar below the heatmap.

Fig. S6. $F_1$ progenies obtained by crosses between the white-flowered cultivar Shirayuki (female) and the green-flowered cultivar Seyshelles (male).

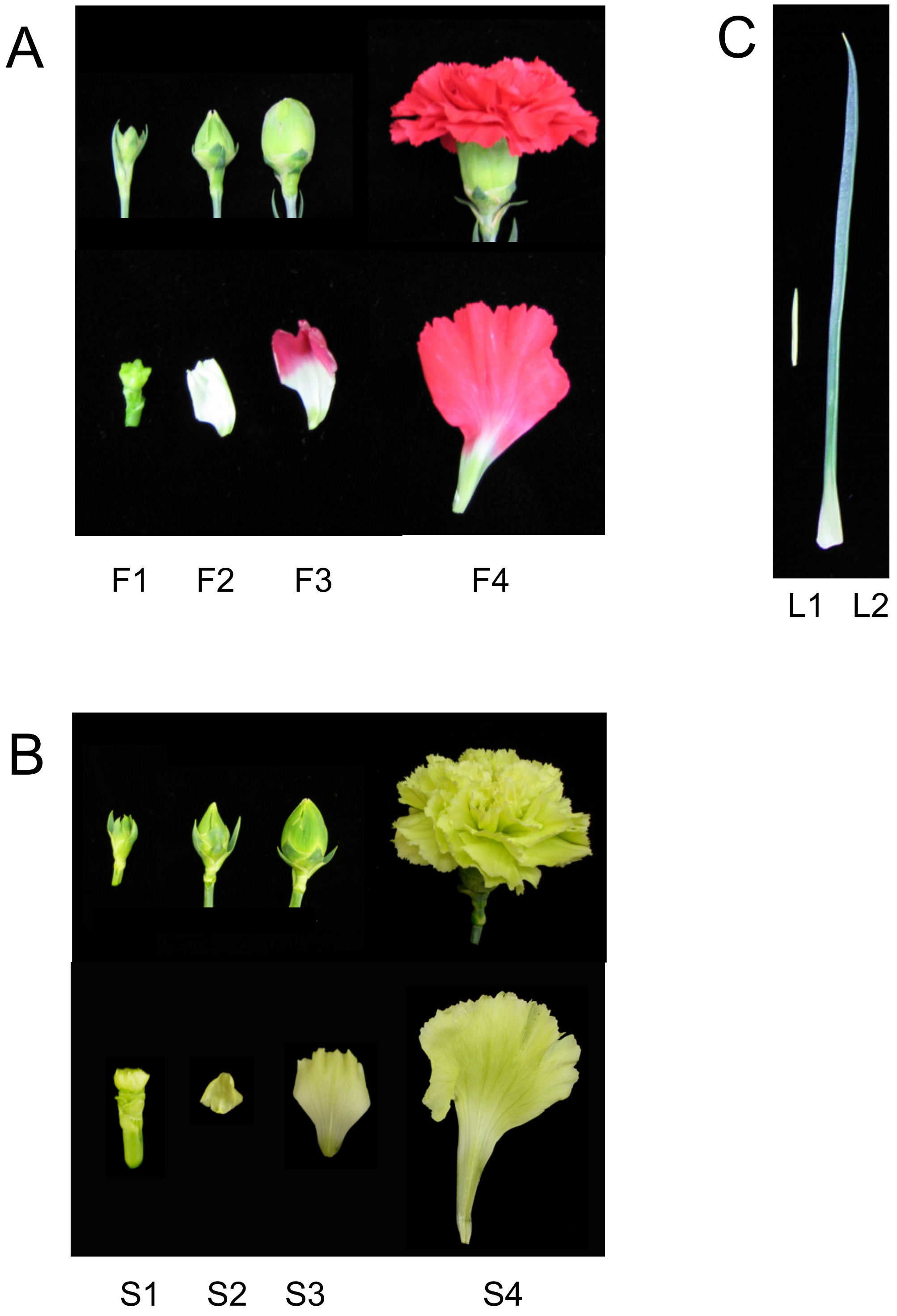


Figure S1

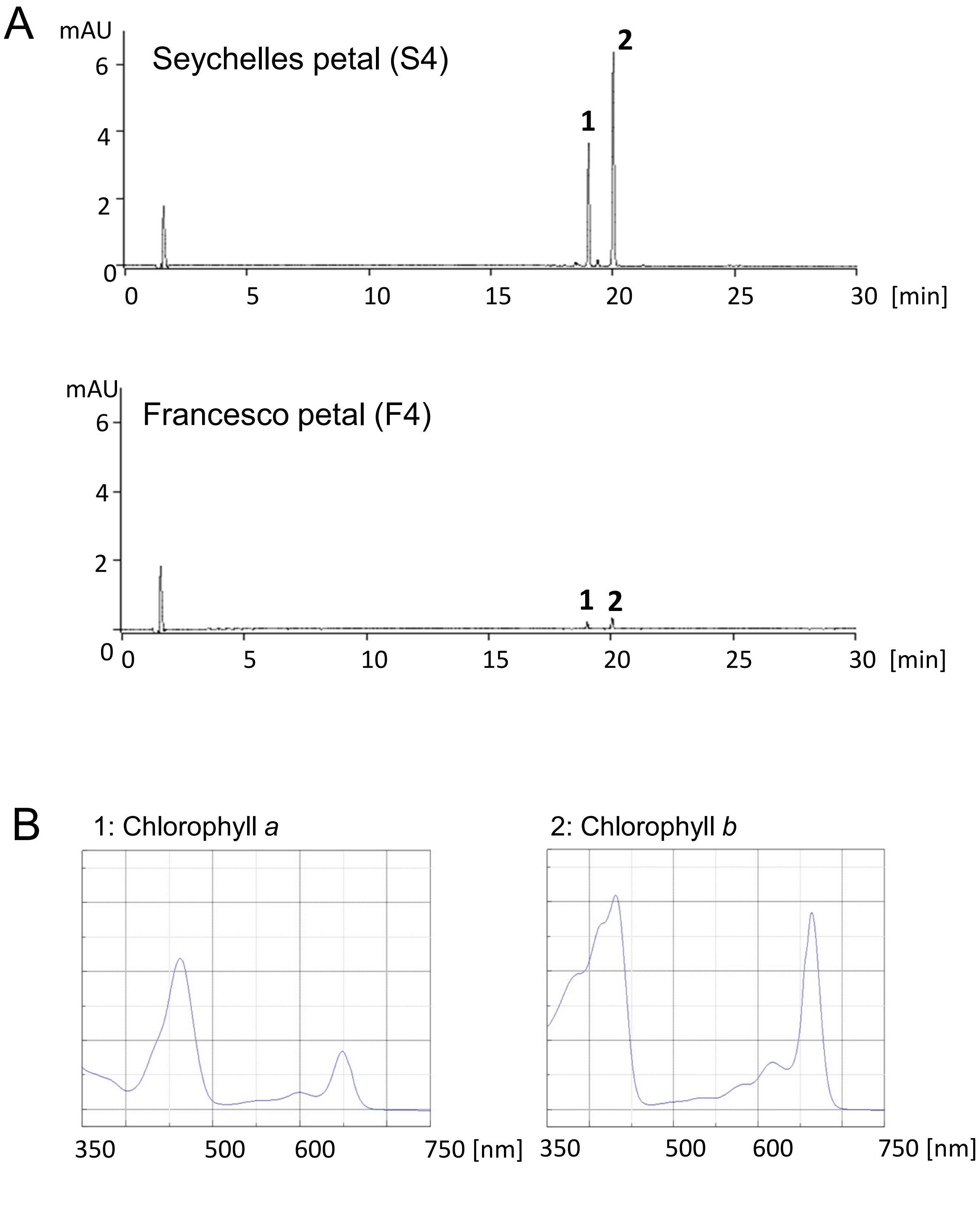
A
mAU
Seychelles petal (S4)
6
4
2
0
1
2
0
5
10
15
20
25
30 [min]
mAU
Francesco petal (F4)
6
4
2
0
1 2
0
5
10
15
20
25
30 [min]
B
1: Chlorophyll *a*
2: Chlorophyll *b*
350
500
600
750 [nm]
350
500
600
750 [nm]

Figure S2

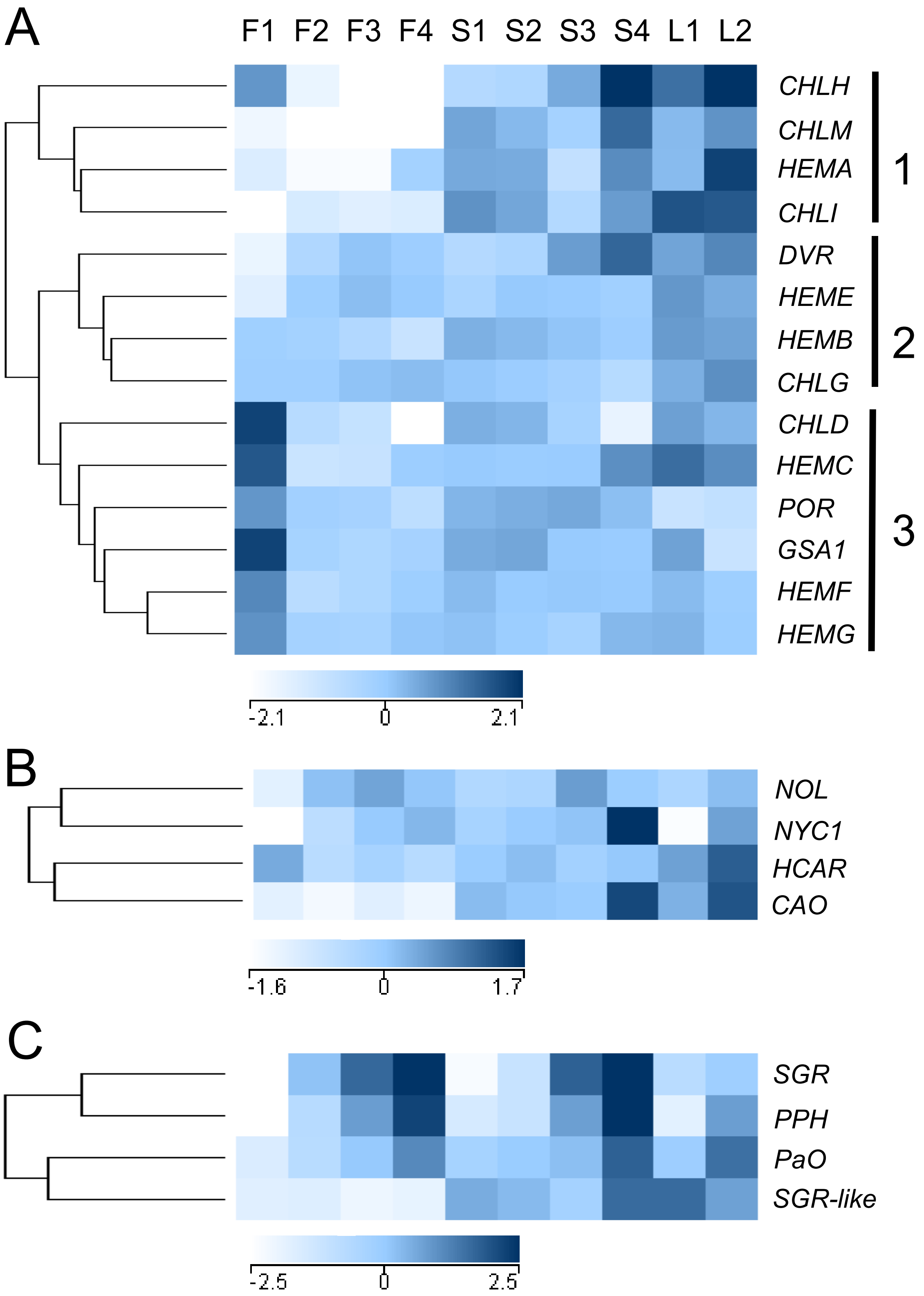


Figure S3A, B, and C

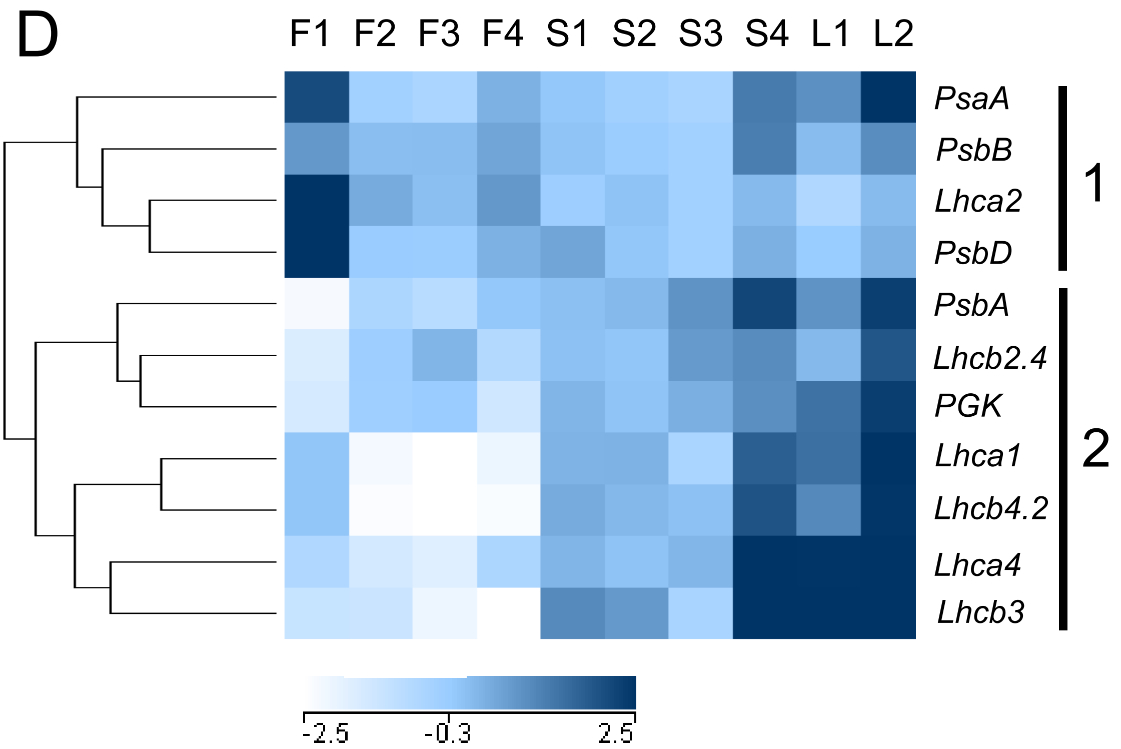
D
F1
F2
F3
F4
S1
S2
S3
S4
L1
L2
PsaA
PsbB
Lhca2
PsbD
PsbA
Lhcb2.4
PGK
Lhca1
Lhcb4.2
Lhca4
Lhcb3
1
2
-2.5
-0.3
2.5

Figure S3D

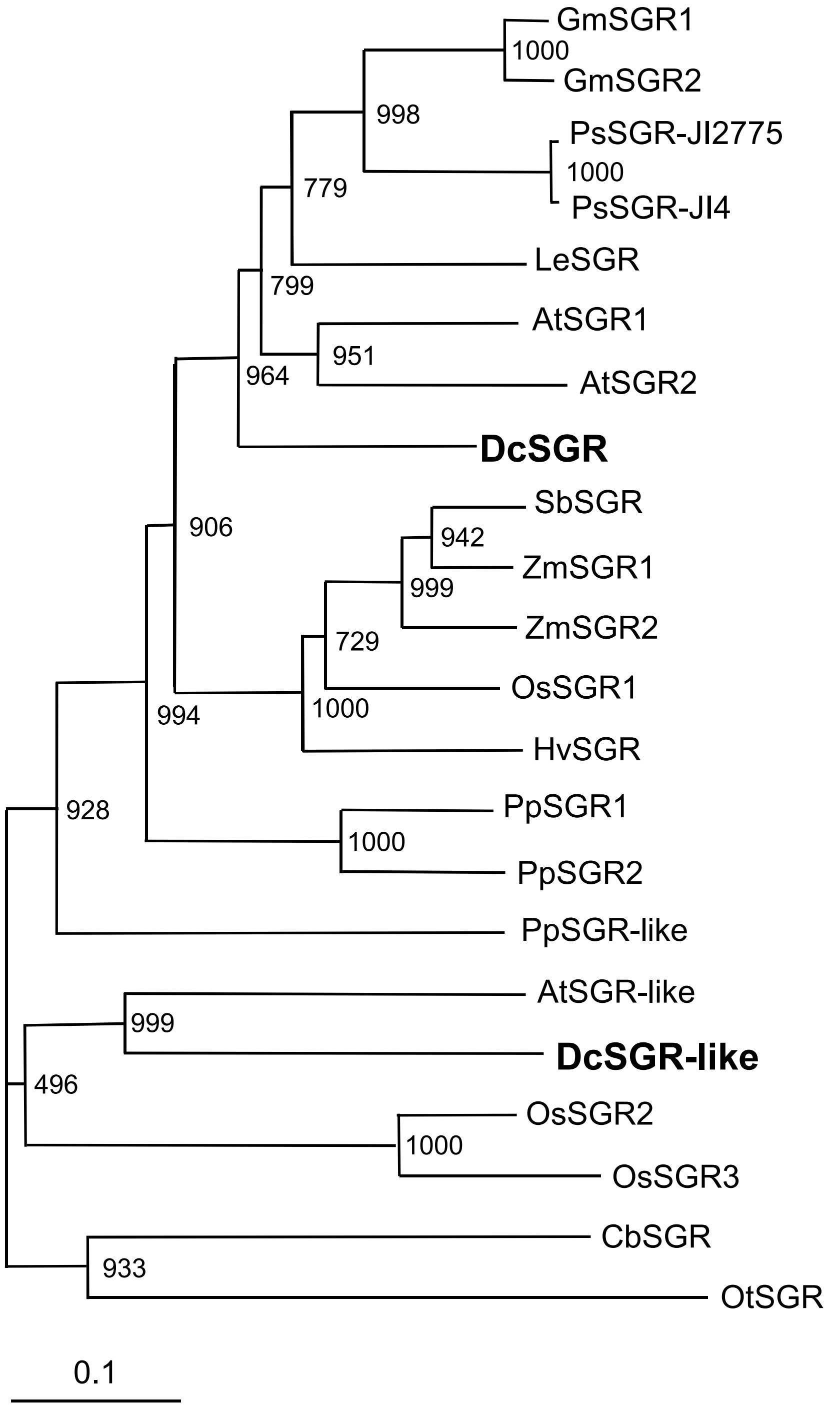
GmSGR1
1000
GmSGR2
998
PsSGR-JI2775
1000
PsSGR-JI4
779
LeSGR
799
AtSGR1
951
964
AtSGR2
DcSGR
SbSGR
906
942
ZmSGR1
999
ZmSGR2
729
OsSGR1
994
1000
HvSGR
PpSGR1
928
1000
PpSGR2
PpSGR-like
AtSGR-like
999
DcSGR-like
496
OsSGR2
1000
OsSGR3
CbSGR
933
OtSGR
0.1

Figure S4

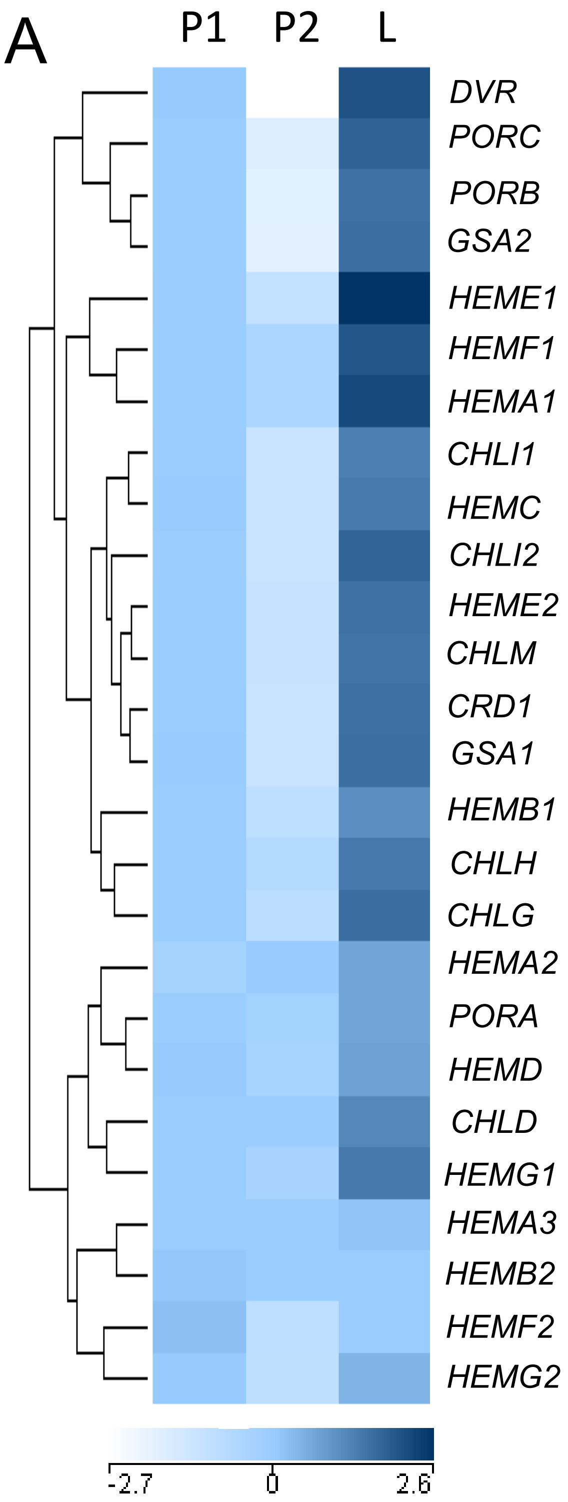
A
P1
P2
L
DVR
PORC
PORB
GSA2
HEME1
HEMF1
HEMA1
CHLI1
HEMC
CHLI2
HEME2
CHLM
CRD1
GSA1
HEMB1
CHLH
CHLG
HEMA2
PORA
HEMD
CHLD
HEMG1
HEMA3
HEMB2
HEMF2
HEMG2
-2.7
0
2.6

Figure S5A

B

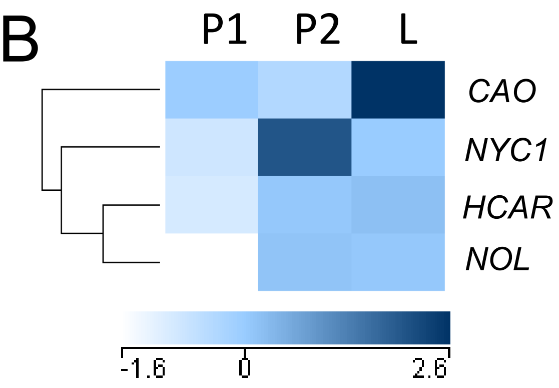


C

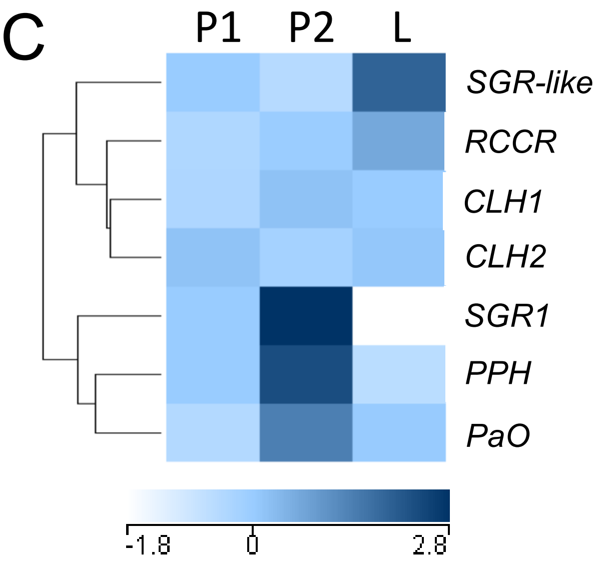


Figure S5B and C

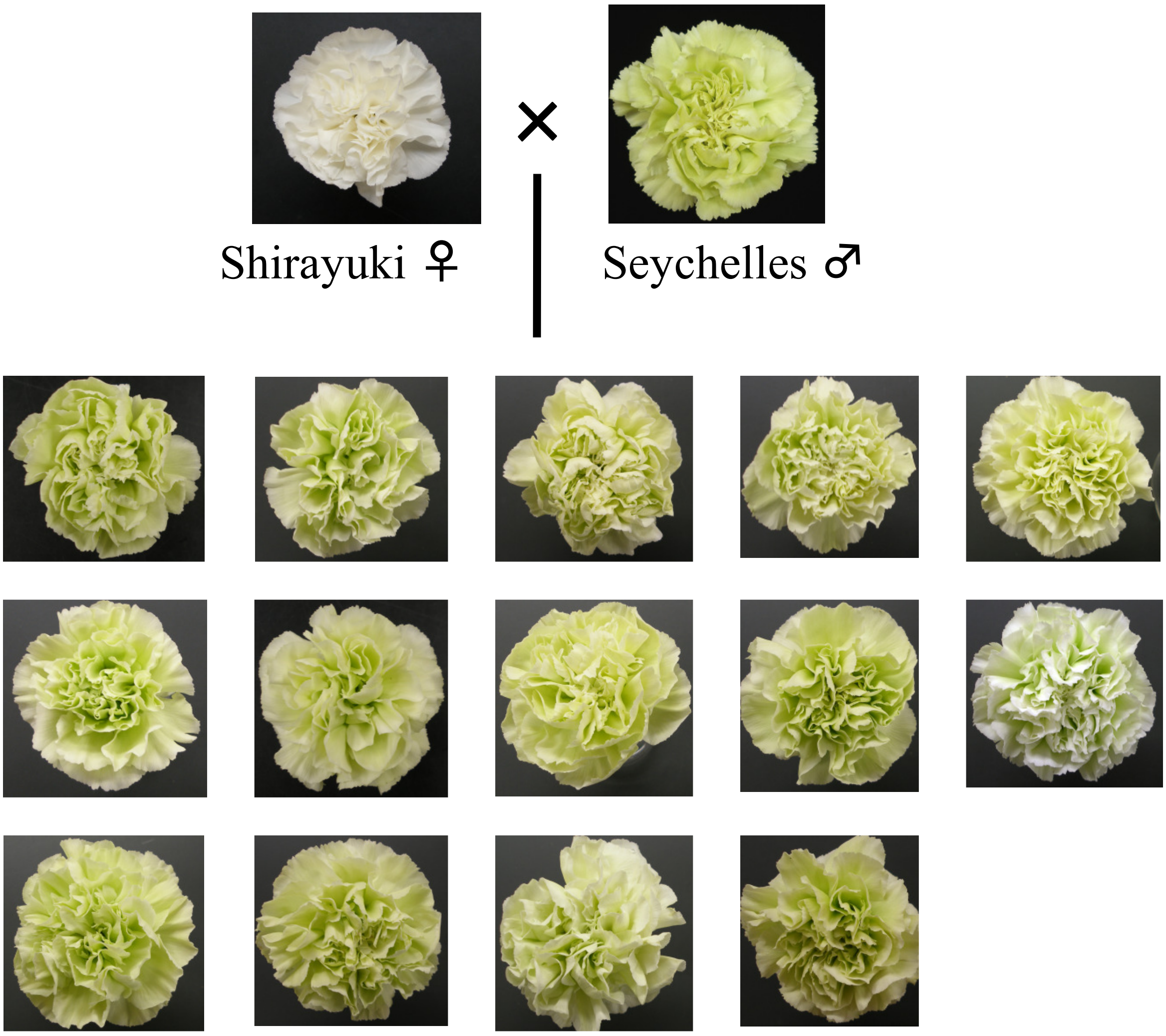


Figure S6

Table S1 Degenerate primers used for cloning

| Gene | Direction | Sequence(5'→3') |
|---|---|---|
| *PPH* | Forward1 | ATGGAAGCCTAAGCTGACAGTNYAYTAYGA |
| | Reverse1 | CAGATAATTAACAACTTCAGGNACYTCRTC |
| | Forward2 | AGTTGGAACTTTTCATTATGARAARCARYT |
| | Reverse2 | ATGTCCAGCAGGAGAAATTTSRTARTANGG |
| *CLH* | Forward1 | GCTTCTCATGGATTTATTGTTGTTGSNCCNCARYT |
| | Reverse1 | CATCATTCCTCTAATTCCTGGAGTATCRTCRTCNADC |
| | Forward2 | CATTCTAGAGGAGGAAAGACTGCTTTYGCNBTNGC |
| | Reverse2 | GAGTATCATCATCCATCATATCCATATGNCCRTARTC |
| *RbcL* | Forward1 | CAGAGACTAAAGCAAGTGTTGGNTTYAARGC |
| | Reverse1 | TGAAGAAGTAGRCCATTATCTCKRCARTARTG |

Partial-length cDNAs encoding PPH, CLH, and RbcL were cloned by RT-PCR using degenerate primers (Forward1 and Reverse1). Nested PCR using Forward2 and Reverse2 primers was performed for cloning of PPH and CLH cDNAs. B, G+T+C; D, G+A+T; K, G+T; N, G+A+T+C; R, A+G; S, G+C; Y, C+T.

Table S1

Table S2 Primers used for RT-qPCR analysis

| Gene | Direction | Sequence (5′→3′) |
|---|---|---|
| *CHLH* | Forward | TCTGCCTGAGGAAGGAGTTGA |
| | Reverse | CGTAGGAGGCTCACCAATGAT |
| *CHLI* | Forward | GCCGAGATGAGGTGACGACA |
| | Reverse | AACGAGCAAACCCGAATCAAT |
| *CHLD* | Forward | ATGTTGATTATGGCCGAAAG |
| | Reverse | TTTCCTGAGATGGCAATACC |
| *CHLM* | Forward | ATGTTGGCTGATGACCGTCCT |
| | Reverse | CAGAAGCCGAGACGACAGCA |
| *NYC1* | Forward | TGTTGTCATCACCTCCCGAAG |
| | Reverse | TTCACAGACATCACACGGAATC |
| *NOL* | Forward | CAAATACCCTCGGTCTGATGAT |
| | Reverse | CAGCAAATCTTGGAGTTGGTCT |
| *HCAR* | Forward | CCTTGGCAGATTTGGTAGTTG |
| | Reverse | CGCTCGTTCCTAATGGTGACA |
| *CAO* | Forward | TTCGCAGAGAAGGTACTGAA |
| | Reverse | CCCGAGTTTGTCATAAGCTA |
| *SGR* | Forward | GATGATGAAGGAAAACACCCA |
| | Reverse | CTACCACCTCGTCTCTTTGC |
| *SGR-like* | Forward | GGTGTATTTCCATTCAAGTTCAA |
| | Reverse | GTTTTCGGGTTTCATTTCCT |
| *CLH* | Forward | ATGGACAAAGGCAAGCAGACG |
| | Reverse | CCAGTGTCGGTATGCCATCG |
| *PPH* | Forward | TGCTCCTTGGTCTGGTGAAGT |
| | Reverse | CCCTCCACAAATCAACAGAGT |
| *PaO* | Forward | CAAAGTTGAATCCAGTGGTCCG |
| | Reverse | TGTGTCTATCTCTATCTTATTGGCG |
| *RbcL* | Forward | TATCTTGGCAGCATTCCGAGTA |
| | Reverse | TACCAGTAGAAGATTCGGCGG |
| *PGK* | Forward | CGGCTGTGAGAGGAGTGGTTT |
| | Reverse | AACATTCAAATCCGCCCTAACA |
| *Actin* | Forward | CACACTGGTGTCATGGTTGG |
| | Reverse | CACAATACCGTGCTCAATTGG |

Table S2

Table S3 Pearson correlation coefficients between chlorophyll content and gene expression level during development of petals and leaves

| Gene | *r* | *P* |
| --- | --- | --- |
| *HEMA* | 0.14 | 0.6961 |
| *CHLH* | 0.74 | 0.0140 * |
| *CHLI* | 0.92 | 0.0002 ** |
| *CHLM* | 0.44 | 0.2080 |
| *NYC1* | 0.34 | 0.3391 |
| *NOL* | 0.37 | 0.2963 |
| *HCAR* | 0.91 | 0.0003 ** |
| *CAO* | 0.43 | 0.2161 |
| *SGR* | -0.18 | 0.6230 |
| *SGR-like* | 0.43 | 0.2158 |
| *CLH* | 0.98 | 0.0001 ** |
| *PPH* | -0.14 | 0.7078 |
| *PaO* | 0.62 | 0.0582 |
| *PsaA* | 0.96 | 0.0001 ** |
| *PsbA* | 0.09 | 0.7998 |
| *Lhcb3* | 0.96 | 0.0001 ** |
| *RbcL* | 0.98 | 0.0001 ** |
| *PGK* | 0.97 | 0.0001 ** |

Chlorophyll content and gene expression levels are presented in Figure 2 and 3, respectively. *, $P < 0.05$; **, $P < 0.01$.

Table S3

Table S4 Pearson correlation coefficients between chlorophyll content and gene expression level in petals of white- and green-flowered cultivars.

| Gene | *r* | *P* | |
|---|---|---|---|
| *HEMA* | 0.35 | 0.3171 | |
| *CHLH* | 0.87 | 0.0010 | ** |
| *CHLI* | 0.66 | 0.0374 | * |
| *CHLM* | 0.54 | 0.1046 | |
| *NYC1* | 0.09 | 0.8074 | |
| *NOL* | -0.26 | 0.4746 | |
| *HCAR* | -0.23 | 0.5167 | |
| *CAO* | 0.15 | 0.6732 | |
| *SGR* | -0.03 | 0.9434 | |
| *SGR-like* | 0.87 | 0.0011 | ** |
| *CLH* | 0.25 | 0.4780 | |
| *PPH* | -0.27 | 0.4425 | |
| *PaO* | -0.08 | 0.8223 | |
| *PsaA* | 0.60 | 0.0693 | |
| *PsbA* | -0.58 | 0.0809 | |
| *Lhcb3* | 0.79 | 0.0069 | ** |
| *RbcL* | 0.85 | 0.0018 | ** |
| *PGK* | 0.82 | 0.0034 | ** |

Chlorophyll content and gene expression levels are presented in Figure 4. *, $P < 0.05$; **, $P < 0.01$.

Table S4
